# Statistical image properties of print advertisements, visual artworks and images of architecture

**DOI:** 10.3389/fpsyg.2013.00808

**Published:** 2013-11-05

**Authors:** Julia Braun, Seyed A. Amirshahi, Joachim Denzler, Christoph Redies

**Affiliations:** ^1^Experimental Aesthetics Group, Institute of Anatomy I, University of Jena School of Medicine, Jena University HospitalJena, Germany; ^2^Computer Vision Group, Friedrich Schiller UniversityJena, Germany

**Keywords:** experimental aesthetics, digital image analysis, self-similarity, complexity, anisotropy, fractal dimension, Fourier spectrum, Pyramid of Histograms of Oriented Gradients (PHOG)

## Abstract

Most visual advertisements are designed to attract attention, often by inducing a pleasant impression in human observers. Accordingly, results from brain imaging studies show that advertisements can activate the brain's reward circuitry, which is also involved in the perception of other visually pleasing images, such as artworks. At the image level, large subsets of artworks are characterized by specific statistical image properties, such as a high self-similarity and intermediate complexity. Moreover, some image properties are distributed uniformly across orientations in the artworks (low anisotropy). In the present study, we asked whether images of advertisements share these properties. To answer this question, subsets of different types of advertisements (single-product print advertisements, supermarket and department store leaflets, magazine covers and show windows) were analyzed using computer vision algorithms and compared to other types of images (photographs of simple objects, faces, large-vista natural scenes and branches). We show that, on average, images of advertisements and artworks share a similar degree of complexity (fractal dimension) and self-similarity, as well as similarities in the Fourier spectrum. However, images of advertisements are more anisotropic than artworks. Values for single-product advertisements resemble each other, independent of the type of product promoted (cars, cosmetics, fashion or other products). For comparison, we studied images of architecture as another type of visually pleasing stimuli and obtained comparable results. These findings support the general idea that, on average, man-made visually pleasing images are characterized by specific patterns of higher-order (global) image properties that distinguish them from other types of images. Whether these properties are necessary or sufficient to induce aesthetic perception and how they correlate with brain activation upon viewing advertisements remains to be investigated.

## Introduction

Neuroeconomics and neuroaesthetics are two areas of experimental aesthetics, which study responses of the human brain to advertisements and beautiful images or objects, respectively. Both types of visual stimuli can induce the experience of pleasantness in human observers. At least in part, they also activate similar regions of the brain's self-reflective and reward circuitries, for example, the medial orbitofrontal cortex, the ventromedial prefrontal cortex, the ventral pallidum and the ventral striatum (Erk et al., 2002; O'Doherty et al., 2003; Cela-Conde et al., 2004; Kawabata and Zeki, 2004; Vartanian and Goel, 2004; Jacobsen et al., 2006; Schaefer et al., 2006; Simmons et al., 2013). Other brain systems that are associated with the perception of visually pleasing stimuli, such as artworks, are involved also in moral judgment (Zaidel and Nadal, 2011; Avram et al., 2013) or belong to the default mode network (Vessel et al., 2012).

Along another line of research in experimental aesthetics, the computational approach aims to identify the statistical properties of visually pleasing images and to relate them to visual perception (Hoenig, 2005; Datta et al., 2006; Li and Chen, 2009; Graham and Redies, 2010; Amirshahi et al., 2012, 2013). For example, recent studies revealed that subsets of artworks possess a scale-invariant Fourier power spectrum (Graham and Field, 2007; Redies et al., 2007a,b; Alvarez-Ramirez et al., 2008). Images of natural scenes also show this property (Field, 1987; Burton and Moorhead, 1987; Field and Brady, 1997; Simoncelli, 2003). Interestingly, the human visual system is adapted to process natural scene statistics efficiently (Parraga et al., 2000; Olshausen and Field, 2004). Taylor and colleagues demonstrated fractal-like structure in both natural scenes and abstract expressionist paintings (Taylor, 2002; Taylor et al., 2011). Based on these similarities, it has been speculated that visually pleasing images follow universal regularities so that they can be processed efficiently by the human visual system (Zeki, 1999; Redies, 2007). In a similar vein, it was proposed that stimuli that can be processed fluently are more aesthetic in general (Reber et al., 2004). In the computational approach, a particular focus was placed on visual artworks and photographs (Datta et al., 2006; Li and Chen, 2009; Amirshahi et al., 2012; Redies et al., 2012), but other types of visually pleasing images, such as graphic novels (Koch et al., 2010) and aesthetic writings (Melmer et al., 2013) have also been studied.

Advertisements are another type of man-made images that attract human attention, often by using pleasant visual stimuli. Many psychological studies have investigated what contents are suited for advertisements in order to evoke a pleasant feeling in the observer, and there are elaborate practical instructions on how to produce an appealing visual layout for print advertisements (Assael et al., 1967; Edell and Staelin, 1983; Finn, 1988; Bushko and Stansfield, 1997). Basic features of print advertisements, such as color, size, and spacing of dominant pictorial and text elements, have been examined (Assael et al., 1967), also in cross-cultural studies (Cutler and Javalgi, 1992). However, to the best of our knowledge, there are no studies to date on higher-order global statistical image properties of print advertisements that may possibly relate to aesthetic perception.

Another source of man-made, visually pleasing stimuli is architecture. The biophilia concept of architecture conjectures that urban planning and architectural design should be based on fractal (self-similar) geometry (Joye, 2007, 2011; Taylor and Sprott, 2008). This theory is based on the observation that humans prefer fractal geometry in their environment (Hagerhall et al., 2004; Taylor et al., 2005), possibly because the natural surroundings of our ancestors had fractal characteristics (“savannah hypothesis”; Orians, 1986; Forsythe et al., 2011). However, Torralba and Oliva (2003) studied simple image statistics, such as Fourier spectral signatures in images of street scenes and buildings, and showed that cardinal (horizontal and vertical) orientations are more prevalent in these images than in images of natural scenes.

In the present work, we investigate higher-order image properties that have been studied in visually pleasing stimuli before. On the one hand, we used a modern computational method that was developed for object recognition and categorization, the Pyramid of Histograms of Oriented Gradients (PHOG) method (Dalal and Triggs, 2005; Bosch et al., 2007). With this method, the following measures were calculated:

(1) *Self-similarity*. Fractal-like image structure can evoke aesthetic experience in humans (see above). Using the PHOG method, it has been shown that museum paintings are self-similar (Amirshahi et al., 2012, 2013; Redies et al., 2012).
(2) *Complexity.* Berlyne (1974) postulated that images of intermediate complexity are considered more aesthetic than simple or highly complex images in general (Berlyne, 1974). Several studies confirm that complexity plays an important role in aesthetic perception (Jacobsen and Hofel, 2002; Rigau et al., 2008; Forsythe et al., 2011; Redies et al., 2012).
(3) *Anisotropy.* This measure describes the statistical variance (heterogeneity) of image features across orientations in an image. For museum paintings and graphic artworks, a low degree of anisotropy was found both for the Fourier spectral profile (Koch et al., 2010; Melmer et al., 2013) and for histograms of oriented gradients (Redies et al., 2012).
(4) *Birkhoff-like measure.* Birkhoff (1933) proposed that the aesthetic appeal of visual stimuli is a function of the ratio of order and complexity in an image. We proposed to substitute order by self-similarity in this ratio (Redies et al., 2012).

On the other hand, the above measures were compared to the following features that have been calculated for visually pleasing images before, also by other groups (for references, see above):

(5) *Slope of log-log plots of radially averaged Fourier power*. For large subsets of monochrome artworks, the slope of the Fourier spectrum is about -2, i.e., the spectra are scale-invariant and the images have a fractal-like structure.
(6) *Fractal dimension*. This measure reflects the density of edges in binarized images and is closely related to complexity (Mureika and Taylor, 2013). In a series of experiments, Taylor and colleagues showed that humans prefer intermediate values for the fractal dimension in both natural and artificial images (Taylor et al., 2011).

Using these six measures, we compared images of advertisements and architecture with various previously studied image categories, including colored artworks of Western provenance, and asked the following questions:

(1) Can the above measures be used to discriminate the diverse image categories?
(2) Given their intended visual appeal, do advertisements and images of architecture share statistical image properties with artworks and complex natural scenes?
(3) In how far do different categories of advertisements vary with respect to the types of products promoted (cars, fashion, cosmetics etc.)?

## Materials and methods

### Image datasets

We investigated 15 different categories of images, focusing on advertisements, artworks and architecture. For comparison, datasets of images that were studied before, including faces, simple objects, and natural scenes and patterns, were analyzed (control images). Each dataset consisted of about 200 color images.

The images of artworks and advertisements (except for show windows) were scanned from high-quality art books and advertisement brochures or magazines, respectively. For scanning, a calibrated digital scanner (Perfection 3200 Photo, Epson, Owa, Japan) was used, as described before (Redies et al., 2012). Images of the other datasets and show windows were taken with a digital camera (EOS 500D with EF- S15- 85 mm f/3.5-5.6 IS USM lens; Canon, Tokyo, Japan) by the authors (Julia Braun and Christoph Redies).

#### Artworks

**Figure 1:**
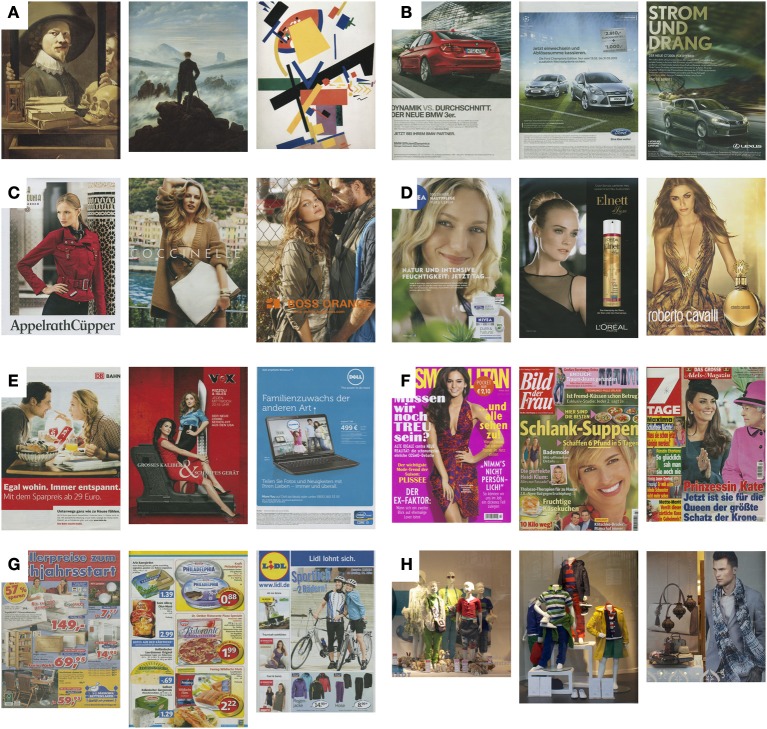
**Examples from the image datasets of artworks and advertisements**. Three images each are shown from the dataset of artworks **(A)**, advertisements for automobiles and accessories **(B)**, fashion **(C)**, cosmetics **(D)**, other products **(E)**, magazine covers **(F)**, supermarket and department store leaflets **(G)**, and show windows **(H)**.

For this category, we selected 197 colored (mostly oil) paintings from a previously analyzed dataset (Redies et al., 2012). The images were selected so that they represented a wide variety of subject matters (21 paintings of architecture, 52 portraits, 23 natural scenes, 60 abstract paintings and 41 other subject matters). The following art periods were covered: Renaissance (20 paintings from 18 artists), Baroque (20 paintings from 18 artists), Romanticism (12 paintings from 9 artists), Realism (20 paintings from 11 artists), Impressionism (20 paintings from 18 artists), Art Nouveau (5 paintings from 3 artists), Expressionism (20 paintings from 9 artists), Fauvism (7 paintings from 4 artists), Cubism (15 paintings from 7 artists), Surrealism (12 paintings from 4 artists), Suprematism (10 paintings from 6 artists), and abstract paintings (36 paintings from 25 artists, including 16 abstract expressionist paintings from 14 artists). Examples are shown in Figure 1A. Images were scanned at a size higher than about 4 million pixels. In the statistical measures analyzed, the sample of 197 paintings did not differ from the previously published dataset (Redies et al., 2012).

#### Advertisements

First, single pages that represented advertisements of one product were scanned from current magazines that were purchased in two newspaper kiosks. An effort was made to include as many different categories of magazines available in the shops. The group of single-product advertisements was further divided into advertisements for cars and automobile accessories (200 images; Figure 1B), fashion (mostly for women, 200 images; Figure 1C), cosmetics (198 images; Figure 1D) and others products (204 images; Figure 1E). Brochures obtained from local car sellers complemented the car image category. Second, other types of advertisements were scanned. They included covers of various womens' and TV magazines (196 images; Figure 1F) and leaflets from supermarkets and department stores with advertisements for grocery, furniture, hardware and other stores (212 images; Figure 1G). Members of the laboratory contributed this material. The cover page, the middle sheet and the rear page of the leaflets were used and typically contained advertisements for several products on each page. In addition, one of the authors (Julia Braun) took 85 photographs of show windows of fashion shops in Jena and Berlin, Germany, with a digital camera, as described above (Figure 1H). Photographs were taken during two walks in major shopping districts. All store windows encountered were photographed, except for those with strong light reflections. The photographs were cropped so that the height of the window fitted the height of the image.

For a general comparison of advertisement images with the other categories of images, we created a dataset that consisted of 30 images randomly selected from each of the seven advertisement subsets described above (210 images in total).

#### Architecture

**Figure 2:**
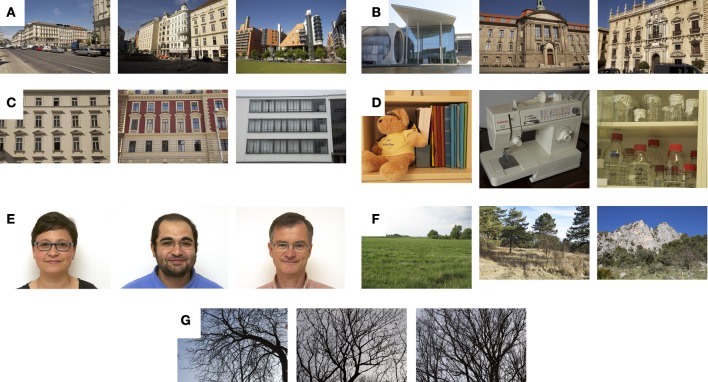
**Examples from the image datasets of architecture and other image categories**. Three images each are shown from the photography dataset of urban scenes **(A)**, buildings **(B)**, facades **(C)**, simple objects **(D)**, faces **(E)**, large-vista natural scenes **(F)**, and branches **(G)**. Because of copyright issues, original face photographs from the AR face database are not shown in **(E)**, but similar photographs of three of the authors.

Photographs of architecture in Austria, Germany and Spain were obtained for three different ranges of distance to the photographed objects. First, 200 photographs of urban scenes, which represent street views or a group of buildings, sometimes with horizon, were taken (Figure 2A). Second, 200 entire buildings were photographed (Figure 2B). Third, 3–4 floors of 175 facades were photographed; an attempt was made not to include cars or people in front of the ground floor (Figure 2C).

For comparison with other types of images, we included previously analyzed datasets (control images; Redies et al., 2012) in the present study. These image datasets are available on the following webpage: www.inf-cv.uni-jena.de/en/aesthetics.

#### Simple objects

This dataset included 200 photographs of ordinary household and laboratory equipment (Figure 2D).

#### Face images

This dataset comprised 200 face photographs of about 100 persons of both genders who were either smiling (72 images) or showed a neutral facial expression (123 images). These photographs were randomly selected from the AR face database (Martinez and Benavente, 1998). Similar photographs are shown in Figure 2E.

#### Natural scenes and patterns

Images of artworks share statistical similarities with images of natural scenes and patterns, in particular large-vista natural scenes (Redies et al., 2007a,b), also in the PHOG analysis (Redies et al., 2012). For comparison, the following datasets were used in the present study: 200 images of large-vista natural scenes of different landscapes (Figure 2F), including the horizon, and 200 images of branches that were taken in winter without foliage (Figure 2G).

### Image calculations

For each image, values for self-similarity, complexity and anisotropy were obtained with the PHOG method, as described before (Amirshahi et al., 2012; Redies et al., 2012). Because halftone dots were visible in a small number of the scanned artworks, image size was reduced to 100,000 pixels by bicubic interpolation. This reduction was carried out for all image categories because the measured values depend on the image size (Redies and Groß, 2013). Color images were transformed into the Lab color space. The general procedure to calculate the PHOG measures is described in the Appendix.

**Figure 3:**
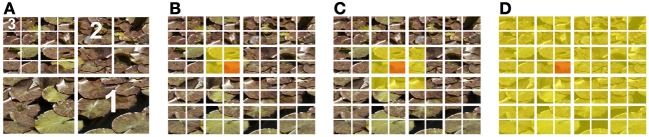
**Different approaches to measure self-similarity**. The construction of the pyramid for the calculation of the histogram of oriented gradients (HOG) features is shown in **(A)**. The numbers in **(A)** indicate the levels of the pyramid. To determine self-similarity, a section at a given level of the pyramid (orange) can be compared to the parent section **(**yellow in **B)**, to the neighboring sections **(**yellow in **C)** or the entire image at the ground level **(**yellow in **D)**.

Three different possibilities to calculate self-similarity were compared (Figure 3). The histograms of each section can be compared to the histograms (i) of the parent section at the previous level (parent approach; Figure 3B), (ii) of all the adjacent (neighboring) sections at the same level (neighbor approach; Figure 3C), (iii) of the entire image at level 0 (ground approach; Figure 3D). Self-similarity values obtained for the three levels of the pyramid (Figure 3A) were averaged, with each level carrying the same weight.

The slope of log-log plots of the radially averaged Fourier power spectrum was determined as described for natural scenes and artworks before (Burton and Moorhead, 1987; Field, 1987; Graham and Field, 2007; Redies et al., 2007a,b). In brief, images were padded according to square ones by adding a uniform border with a gray level that was equal to the mean gray level of the image. All images were reduced to a size of 1024 × 1024 pixels by bicubic interpolation and isotropic scaling. A discrete Fourier transform (2d Fast Fourier Transform) was calculated to obtain the 2d power spectrum, which was then transformed into a 1d spectrum by rotational averaging for each frequency. In the log-log plane, power was plotted as a function of spatial frequency. To measure the slope of the resulting frequency spectrum, a least-squares fit of a line was performed in the range of 10–256 cycles/image and the slope of the line was determined.

The fractal dimension can be seen as an indicator for the complexity of a pattern: A high fractal dimension indicates high complexity, while a low fractal dimension indicates low complexity (Mureika and Taylor, 2013). The fractal dimension was estimated with the box-counting method, as described by Hagerhall et al. (2004). Because the box-counting method requires binarized images, we applied the canny-edge filter (Canny, 1986) to each image. Next, each image was covered by a mesh of “boxes” that represented equally sized squares. This procedure was repeated for decreasing box sizes ε, which results in an increasingly finer mesh. Let *N*(ϵ) be the number of boxes that are occupied by the pattern in relation to a specific box size ϵ. According to the power law relation *N*(ϵ) ~ ϵ^−*D*^, the box-counting dimension *D* can be estimated by fitting a line to the plot log *N*(ϵ) vs. log (1/ϵ) and measuring the slope of this line. All calculations were performed with the MatLab program.

### Statistical analysis

For the statistical verification, we carried out the non-parametric Wilks-Lambda multivariate analysis of variance test on all 15 image categories with all the six measurements, followed by the Tukey *post-hoc* test for individual comparisons for all pairs of categories.

## Results

We measured statistical properties in 15 different categories of images, with a particular focus on advertisements, artworks and architecture. Six features that were previously studied in visually pleasing images were calculated (self-similarity, complexity, anisotropy, the Birkhoff-like measure, the slope of the 1d Fourier spectrum, and the fractal dimension; see Introduction). Statistical testing showed overall differences between all 15 groups and all six measures (*p* < 0.001).

**Figure 4:**
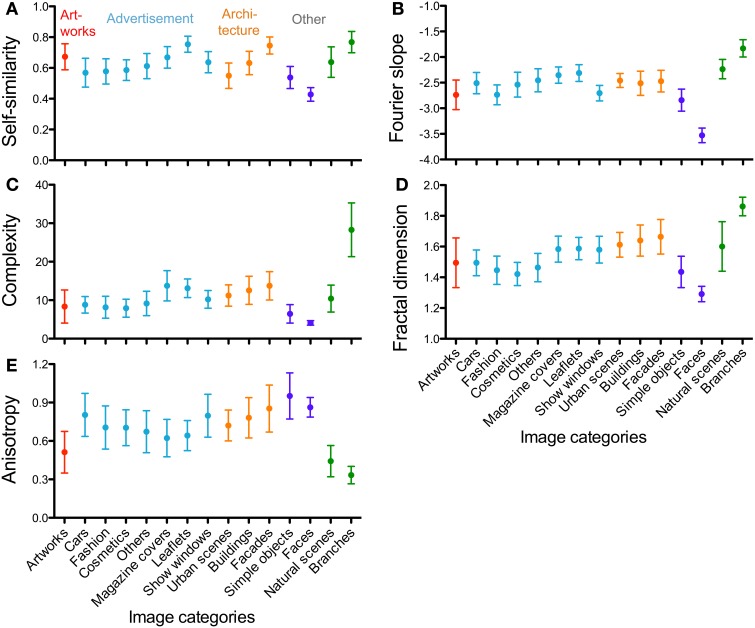
**Whisker plots of the means for self-similarity **(A)**, Fourier slope (B), complexity (C), fractal dimension **(D)** and anisotropy (E)**. The image categories are indicated below the y-axis in panels **(D,E)** for all panels. The colors represent the different image categories (red, artworks; light blue, advertisement; orange, architecture; dark blue and green, other image categories). Whiskers represent means ±1 SD.

**Figure 5:**
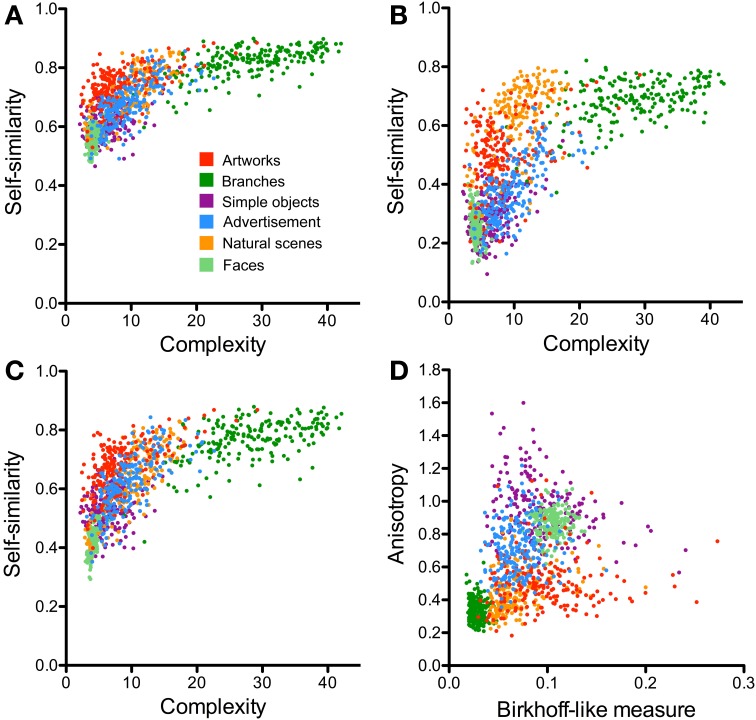
**Scatter plots of the PHOG-derived measures for the different image categories**. **(A–C)** compares results for the three different approaches to calculate self-similarity [parent approach **(A)**, neighbor approach **(B)**, and ground approach **(C)**; see Materials and Methods]. Self-similarity is plotted as a function of complexity. **(D)** Shows a plot of anisotropy vs. the Birkhoff-like measure for the ground approach. Each dot represents one image. The image categories are illustrated by the different colors, as indicated in panel **(A)**.

Median values of the measured properties for all 15 image categories are provided in Tables 1–5 and are summarized in Figure 4. Figures 5, 7A compare the results for advertisements with five datasets of image categories that were analyzed previously by our group (artworks and photographs of branches, simple objects, natural scenes and passport-type face photographs; Redies et al., 2012). Results show that images of advertisements can be characterized by a specific combination of the measured properties on average, as discussed in more detail below (section Comparison of Advertisements to Other Image Categories). The other image categories can also be characterized by specific combinations of the six measures (Redies et al., 2012).

**Table 1:** **Comparison of different approaches to calculate self-similarity**.

**Image dataset**	**Self-similarity [median (mean ± SD)]**		
	**Parent approach**	**Neighbor approach**	**Ground approach**
Advertisement (*n* = 210)	0.68 (0.68 ± 0.07)	0.37 (0.38 ± 0.10)	0.62 (0.62 ± 0.09)
Artworks (*n* = 197)	0.74 (0.73 ± 0.06)^*^	0.50 (0.50 ± 0.11)^*^	0.68 (0.67 ± 0.09)^*^
Simple objects (*n* = 200)	0.62 (0.62 ± 0.05)^*^	0.28 (0.29 ± 0.08)^*^	0.53 (0.54 ± 0.07)^*^
Faces (*n* = 200)	0.56 (0.56 ± 0.04)^*^	0.26 (0.26 ± 0.05)^*^	0.43 (0.43 ± 0.04)^*^
Natural scenes (*n* = 200)	0.73 (0.73 ± 0.07)^*^	0.65 (0.62 ± 0.12)^*^	0.64 (0.64 ± 0.10)
Branches (*n* = 200)	0.83 (0.82 ± 0.05)^*^	0.69 (0.69 ± 0.07)^*^	0.78 (0.77 ± 0.07)^*^

* Significantly different from advertisement (p < 0.001).

In the following sections, we will first evaluate the three different methods to calculate self-similarity by the PHOG method. Second, we will compare results for advertisements with the previously studied image categories. This comparison was of interest in particular with respect to man-made images vs. natural images and for images that differ in their degree of their aesthetic appeal. Third, we will compare results obtained for the different types of advertisements. Fourth, results for images of architecture will be described.

### Methodological considerations

The values that are derived from the PHOG method critically depend on several parameters, for example, the resolution of the images and the level of the image pyramid, on which the analysis was performed (Amirshahi et al., 2012). For this reason, we carried out PHOG calculations for all images at the same resolution (100,000 pixels) and at the same level (level 3).

To calculate self-similarity, three different approaches were considered in the present work (see Materials and Methods, Section Image Calculations; Table 1). In two earlier studies from our group, self-similarity was calculated based on the parent approach (Figure 3B; Amirshahi et al., 2012; Redies et al., 2012). The average self-similarity for advertisements assumes intermediate values (0.68; Figure 5A). Values for artworks (0.74), natural scenes (0.73) and branches (0.83) are higher (*p* < 0.001) whereas values for faces (0.56) and simple objects (0.62) are lower (*p* < 0.001). These values are similar to those from our earlier study (Redies et al., 2012).

For the second method of calculating self-similarity (neighbor approach; Figure 3C), overall results follow the same pattern, but self-similarity values were generally lower than for the parent approach (Figures 5A,B, Table 1). This decrease is especially prominent for images of advertisements (0.37), artworks (0.50), faces (0.26) and simple objects (0.28). The decrease of average values for natural scenes (0.65) and branches (0.69) is smaller.

Results for the ground approach (Figures 3D, 5C, Table 1) are intermediate between those of the parent approach and the neighbor approach. Again, the overall pattern of differences is similar compared to the two other approaches.

In conclusion, these results demonstrate that the different PHOG-derived approaches to calculate self-similarity are relatively robust with regard to relative differences between image categories, although absolute values differ. In the following comparison, we chose the ground approach.

### Comparison of advertisements to other image categories

The scatter plots shown in Figures 5C,D, 7A compare the results for advertisements and the other image categories. The plot of self-similarity vs. complexity (Figure 5C), of anisotropy vs. the Birkhoff-like measure (Figure 5D) and Fourier slope vs. fractal dimension (Figure 7A) reveal distinct but partially overlapping clusters for each image category.

For advertisements, self-similarity values (0.62; Figure 4C, Table 2) differ significantly from artworks (0.68, *p* < 0.001), simple objects (0.53, *p* < 0.001), faces (0.43, *p* < 0.001) and branches (0.78, *p* < 0.001) but not from natural scenes (0.64). Complexity values (Figure 4C) obtained for advertisements (9.00) are higher than values for artworks (7.23, *p* < 0.05), simple objects (6.18; *p* < 0.001), and faces (3.99; *p* < 0.001), but much lower than for branches (28.25; *p* < 0.001). The complexity of natural scenes (10.63; Table 2) is similar to that of advertisements. This pattern of differences is similar to the previously published results (Redies et al., 2012) although absolute values differ because of the resolution of the images used (1 million pixels vs. 100,000 pixels) and the approaches to calculate self-similarity varied (see above).

**Table 2:** **Average values for the four PHOG-derived image properties**.

**Image dataset**	**Self-similarity**	**Complexity**	**Anisotropy (× 10^−3^)**	**Birkhoff-like measure**
Artworks (*n* = 197)	0.68 (0.67 ± 0.09)^c^, ^d^	7.23 (8.33 ± 4.30)^a^, ^d^	0.49 (0.51 ± 0.16)^c^, ^d^	0.09 (0.10 ± 0.04)^c^, ^d^
Advertisement^e^ (*n* = 210)	0.62 (0.62 ± 0.09)	9.00 (9.73 ± 3.66)	0.70 (0.71 ± 0.16)^d^	0.07 (0.07 ± 0.02)
Urban scenes (*n* = 200)	0.56 (0.55 ± 0.08)^c^, ^d^	11.28 (11.21 ± 2.77)^b^	0.72 (0.72 ± 0.12)^d^	0.05 (0.05 ± 0.01)^c^, ^d^
Buildings (*n* = 200)	0.63 (0.63 ± 0.08)	12.44 (12.56 ± 3.65)^c^, ^d^	0.75 (0.78 ± 0.16)^c^, ^d^	0.05 (0.05 ± 0.02)^c^, ^d^
Facades (*n* = 175)	0.76 (0.75 ± 0.06)^c^, ^d^	13.80 (13.75 ± 3.68)^c^, ^d^	0.83 (0.85 ± 0.18)^c^, ^d^	0.05 (0.06 ± 0.02)^c^, ^d^
Simple objects (*n* = 200)	0.53 (0.54 ± 0.07)^c^, ^d^	6.18 (6.46 ± 2.40)^c^, ^d^	0.93 (0.95 ± 0.18)^c^, ^d^	0.09 (0.09 ± 0.03)^c^, ^d^
Faces (*n* = 200)	0.43 (0.43 ± 0.04)^c^, ^d^	3.99 (4.09 ± 0.58)^c^, ^d^	0.86 (0.86 ± 0.08)^c^, ^d^	0.11 (0.11 ± 0.01)^c^, ^d^
Natural scenes (*n* = 200)	0.64 (0.64 ± 0.10)	10.63 (10.41 ± 3.51)	0.42 (0.44 ± 0.12)^c^	0.06 (0.07 ± 0.02)
Branches (*n* = 200)	0.78 (0.77 ± 0.07)^c^, ^d^	28.25 (28.30 ± 6.99)^c^, ^d^	0.33 (0.33 ± 0.07)^c^, ^d^	0.03 (0.03 ± 0.01)^c^, ^d^

Values represent median (mean ± SD), calculated on the basis of the ground approach.

a, b, c Significantly different from advertisement (^a^p < 0.05; ^b^p < 0.01; ^c^p < 0.001).

d Significantly different from natural scenes (p < 0.001).

e Images were randomly sampled from all seven advertisement subcategories.

We obtained distinct clusters for each image category in the scatter plot of anisotropy vs. the composite Birkhoff-like measure (Figure 5D, Table 2). For example, compared to advertisements (0.70 × 10^−3^), anisotropy is lower for artworks (0.49 × 10^−3^, *p* < 0.001), natural scenes (0.42 × 10^−3^, *p* < 0.001) and branches (0.33 × 10^−3^, *p* < 0.001), whereas it is higher for simple objects (0.93 × 10^−3^, *p* < 0.001) and faces (0.86 × 10^−3^, *p* < 0.001). For the Birkhoff-like measure, values are similar for advertisements and natural scenes (Figure 5D, Table 2). The comparison to all other image categories yields significantly different values.

The Fourier slope values of images of advertisements (−2.57) and architecture (−2.45 to −2.53) are similar (Table 3). Artworks have slope values (−2.77) lower than advertisements (−2.77; *p* < 0.001). Images of natural scenes and branches have slope values close to −2 (Figure 7A) while face images have much lower values than advertisements (−3.51; *p* < 0.001; Figure 7A). For the fractal dimension, as expected, the overall pattern of values is similar to that of complexity (Figures 4C,D).

**Table 3:** **Average values for the Fourier slope and fractal dimension**.

**Image dataset**	**Fourier slope**	**Fractal dimension**
Artworks (*n* = 197)	−2.77 (−2.74 ± 0.29)^c^, ^e^	1.49 (1.49 ± 0.16)^e^
Advertisement (*n* = 210)	−2.57 (−2.54 ± 0.24)^e^	1.51 (1.50 ± 0.12)
Urban scenes (*n* = 200)	−2.45 (−2.46 ± 0.14)^b^, ^e^	1.62 (1.61 ± 0.08)^c^
Buildings (*n* = 200)	−2.53 (−2.51 ± 0.24)^e^	1.65 (1.64 ± 0.10)^c^, ^d^
Facades (*n* = 175)	−2.47 (−2.47 ± 0.21)^a^, ^e^	1.68 (1.66 ± 0.11)^c^, ^e^
Simple objects (*n* = 200)	−2.83 (−2.84 ± 0.22)^c^, ^e^	1.44 (1.44 ± 0.10)^c^, ^e^
Faces (*n* = 200)	−3.51 (−3.53 ± 0.14)^c^, ^e^	1.29 (1.29 ± 0.05)^c^, ^e^
Natural scenes (*n* = 200)	−2.21 (−2.24 ± 0.19)^c^	1.61 (1.60 ± 0.16)^c^
Branches (*n* = 200)	−1.81 (−1.83 ± 0.17)^c^, ^e^	1.87 (1.86 ± 0.06)^c^, ^e^

Values represent median (mean ± SD).

a, b, c Significantly different from advertisement (^a^p < 0.05; ^b^p < 0.01; ^c^p < 0.001).

d, e Significantly different from natural scenes (^d^p < 0.05; ^e^p < 0.001).

### Comparison of different advertisement categories

**Figure 6:**
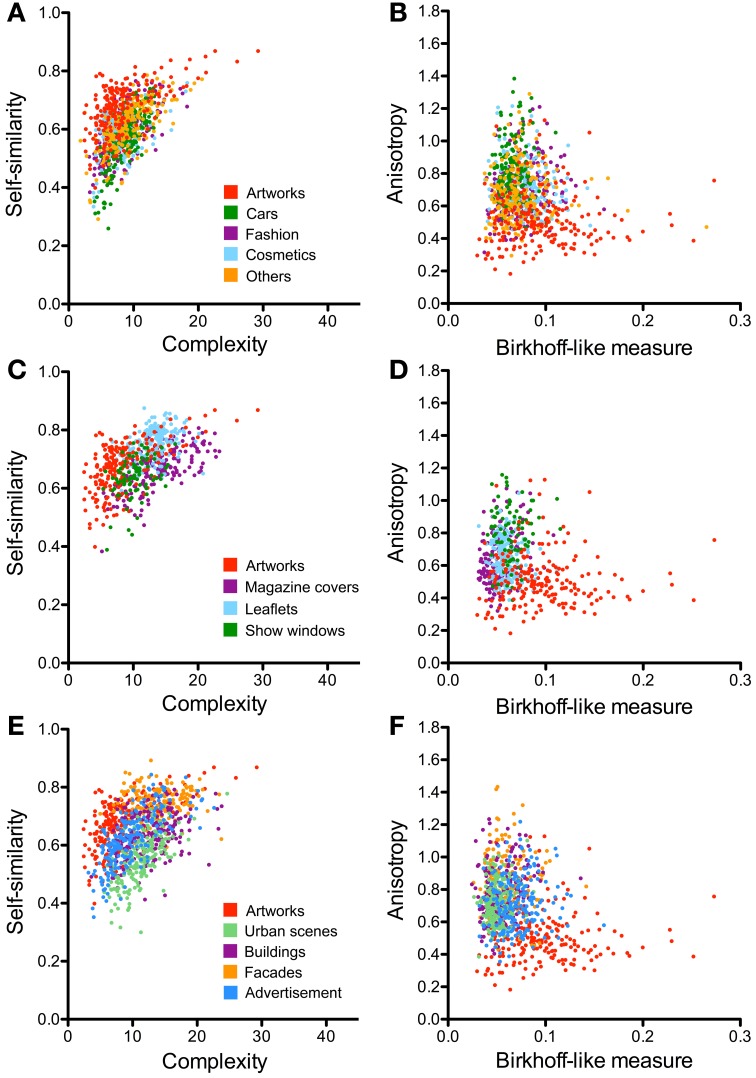
**Scatter plots of the PHOG-derived measures for the different advertisement categories (A–D) and architecture categories (E,F)**. Scatter plots of self-similarity vs. complexity are shown in **(A,C,E)** and of anisotropy vs. the Birkhoff-like measure in **(B,D,F)**. Each dot represents one image. For comparison, results for artworks are represented by the red dots. The image categories are illustrated by the different colors, as indicated in panels (in **A**, for **A** and **B**; in **C**, for **C** and **D**; and in **E**, for **E** and **F**).

**Figure 7:**
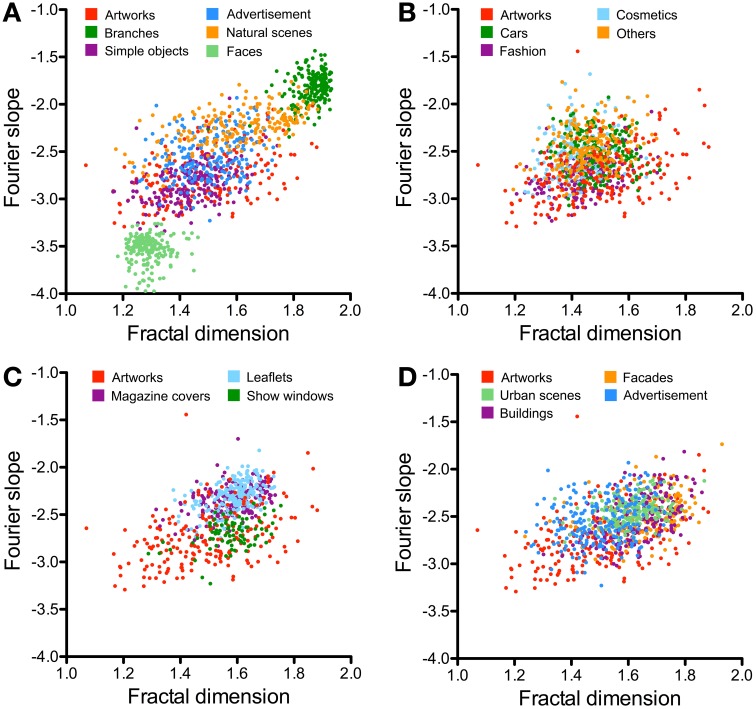
**Scatter plots of the Fourier slope values and the fractal dimension for the different image categories**. **(A)** Compares results for previously studied image categories and artworks. Results for single-product advertisements are shown in **(B)**, for other advertisements in **(C)**, and for architecture in **(D)**. The image categories are illustrated by the different colors, as indicated in each panel.

Figures 6, 7B–D illustrate the results for single-product advertisements (Figures 6A,B, 7B) and the other types of advertisement (magazine covers, supermarket and department store leaflets, and show windows; Figures 6C,D, 7C). Detailed results are listed in Tables 4, 5 and compared to the other image categories in Figure 4.

**Table 4:** **Comparison of different types of advertisements**.

**Image dataset**	**Self-similarity**	**Complexity**	**Anisotropy (× 10^−3^)**	**Birkhoff-like measure**
Advertisement, all (*n* = 1295)	0.63 (0.63 ± 0.10)^c^	9.61 (10.17 ± 3.63)^c^	0.68 (0.70 ± 0.16)^c^, ^f^	0.06 (0.07 ± 0.02)^c^
Advertisement, subset (*n* = 210)	0.62 (0.62 ± 0.09)^c^	9.00 (9.73 ± 3.66)^b^	0.70 (0.71 ± 0.16)^c^, ^f^	0.07 (0.07 ± 0.02)^c^
Cars (*n* = 200)	0.58 (0.57 ± 0.09)^c^, ^f^	8.73 (8.81 ± 2.16)^f^	0.78 (0.80 ± 0.17)^c^, ^f^	0.07 (0.07 ± 0.01)^c^
Fashion (*n* = 200)	0.58 (0.58 ± 0.08)^c^, ^f^	7.64 (8.16 ± 2.84)^f^	0.69 (0.71 ± 0.17)^c^, ^f^	0.08 (0.08 ± 0.02)^c^, ^e^
Cosmetics (*n* = 198)	0.60 (0.59 ± 0.07)^c^, ^f^	7.48 (7.92 ± 2.34)^f^	0.68 (0.70 ± 0.14)^c^, ^f^	0.08 (0.08 ± 0.02)^c^, ^f^
Others (*n* = 204)	0.62 (0.61 ± 0.08)^c^	8.96 (9.16 ± 3.17)^d^	0.65 (0.67 ± 0.16)^c^, ^f^	0.07 (0.07 ± 0.03)^c^
Magazine covers (*n* = 196)	0.67 (0.67 ± 0.07)^e^	13.46 (13.75 ± 3.93)^c^, ^f^	0.59 (0.62 ± 0.15)^c^, ^f^	0.05 (0.05 ± 0.01)^c^, ^f^
Leaflets (*n* = 212)	0.76 (0.75 ± 0.06)^c^, ^f^	13.34 (13.10 ± 2.41)^c^, ^f^	0.63 (0.64 ± 0.12)^c^, ^f^	0.06 (0.06 ± 0.01)^c^, ^e^
Show windows (*n* = 85)	0.64 (0.64 ± 0.07)^a^	10.15 (10.23 ± 2.31)^b^	0.80 (0.80 ± 0.17)^c^, ^f^	0.06 (0.06 ± 0.01)^c^

Values represent median (mean ± SD), calculated on the basis of the ground method.

a,b,c Significantly different from artworks (^a^p < 0.05; ^b^p < 0.01; ^c^p < 0.001; Table 2)

d, e, f Significantly different from natural scenes (^d^p < 0.05; ^e^p < 0.01; ^f^p < 0.001; Table 2).

**Table 5:** **Comparison of different types of advertisements**.

**Image dataset**	**Fourier slope**	**Fractal dimension**
Advertisement (*n* = 1295)	−2.49 (−2.50 ± 0.24)^c^	1.50 (1.51 ± 0.12)^c^
Cars (*n* = 200)	−2.50 (−2.51 ± 0.21)^a^, ^c^	1.48 (1.49 ± 0.08)^c^
Fashion (*n* = 200)	−2.74 (−2.74 ± 0.19)^c^	1.44 (1.45 ± 0.09)^a^, ^c^
Cosmetics (*n* = 198)	−2.55 (−2.54 ± 0.24)^a^, ^c^	1.42 (1.42 ± 0.08)^a^, ^c^
Others (*n* = 204)	−2.48 (−2.45 ± 0.23)^a^, ^c^	1.46 (1.46 ± 0.09)^c^
Magazine covers (*n* = 196)	−2.33 (−2.35 ± 0.16)^a^, ^c^	1.59 (1.58 ± 0.08)^a^
Leaflets (*n* = 212)	−2.30 (−2.31 ± 0.16)^a^, ^b^	1.60 (1.59 ± 0.07)^a^
Show windows (*n* = 85)	−2.70 (−2.70 ± 0.15)^c^	1.58 (1.58 ± 0.09)^a^

Values represent median (mean ± SD).

a Significantly different from artworks (p < 0.001; Table 3)

b, c Significantly different from natural scenes (^b^p < 0.05; ^c^p < 0.001; Table 3).

Compared to the image categories described in the previous section, the dot clusters of the individual advertisement categories overlap to a much larger degree with each other and also with the artworks cluster (Figures 6A–D, 7B,C). Single-product advertisements (cars, fashion, cosmetics and others) tend to have lower complexity, fractal dimension, and self-similarity than the other types of advertisements whereas anisotropy and the Fourier slope tend to be more variable in general (Figure 4, Tables 4, 5). Compared to artworks, single-product advertisements tend to be less self-similar (*p* < 0.001) and more anisotropic (*p* < 0.001) but they do not differ in average complexity (Figures 6A,B, Table 4). Values for the Birkhoff-like measure are lower than for artworks (*p* < 0.001; Figure 6B). The Fourier slope values for single-product advertisements are higher than for artworks (*p* < 0.001), except for fashion images. The fractal dimension is smaller for fashion (1.44, *p* < 0.001) and cosmetics (1.42, *p* < 0.001) than for artworks (1.49) (Figure 7B). The other types of advertisement are more complex (*p* < 0.05 to *p* < 0.001), more anisotropic (*p* < 0.001) and have a lower Birkhoff-like measure and a higher fractal dimension than artworks (*p* < 0.001; Figures 6C,D, 7C, Tables 4, 5). Self-similarity for magazine covers is as high as for artworks, higher for leaflets (*p* < 0.001) and lower for show windows (*p* < 0.05; Figures 4, 6C).

### Images of architecture

The images of architecture (urban scenes, buildings, and facades) are more complex than artworks and single-product advertisements (*p* < 0.001; Figures 4C, 6E; Table 2) but tend to be similar to the other types of advertisements. A similar trend is observed for the fractal dimension (Figures 4D, 7D, Tables 3, 5). The degree of anisotropy resembles that of single-product advertisements and show windows but is higher than for artworks (*p* < 0.001; Figure 6F, Table 2). Self-similarity for buildings is as high as for the combined dataset of advertisements; it is higher for facades (*p* < 0.001) and lower for urban scenes (*p* < 0.001; Figure 6E, Table 2). The Birkhoff-like measure is lower for architecture than for artworks (Figure 6F; *p* < 0.001). The Fourier slope for images of architecture is similar to that of single-product advertisements (Figures 4D, Tables 3, 5) and artworks (Figure 7D).

## Discussion

In this work, we studied statistical image properties in images of advertisements and architecture, and compared them to results of other man-made, visually pleasing images, such as artworks (Graham and Redies, 2010; Redies et al., 2012; Amirshahi et al., 2013; Melmer et al., 2013). Given the similarities in brain responses to these different types of rewarding stimuli (see Introduction), we speculated that the images might also share structural features at the stimulus level. This notion was challenged by measuring image features that have been studied in visually pleasing stimuli before (self-similarity, complexity, anisotropy, slope of the radially averaged Fourier spectrum, and fractal dimension; see Introduction). A particular focus of our study was the question of whether the images of advertisements differ from the other image categories.

In the following paragraphs, we will first point out similarities and differences between advertisements and the other image categories, such as artworks and large-vista natural scenes. Second, we will address the question of whether the measured values relate to the content and layout of different types of advertisements. Third, we will compare images of architecture to the other types of visual stimuli.

### Differences between images of advertisements and other image categories

In an earlier study, we showed that the PHOG measures (self-similarity, complexity and anisotropy) allow distinguishing artworks from many other image categories on average (Amirshahi et al., 2012; Redies et al., 2012). In the present work, we add images of advertisements to this comparison (Figure 5). In addition, we compare the results with previously obtained measurements of the Fourier slope and the fractal dimension (Figure 7).

Our results indicate that, like the other image categories, advertisements can be characterized by a specific combination of these measures (Figures 4, 5C,D, 7A, Tables 2, 3). The results for the combined dataset of advertisements largely resemble those of artworks and natural scenes, although some differences were observed. All three image categories have relatively high values for self-similarity and intermediate values for complexity and the fractal dimension, compared to, for example, photographs of faces and branches. This finding supports the notion that subsets of visually pleasing images share specific statistical properties in general. However, we note that the different measures are not independent. For all images analyzed together, Table 6 provides the Spearman correlation coefficients for the measures. As expected, the strongest correlation is found between complexity and the fractal dimension. Other correlations, e.g., between complexity and self-similarity, are also relatively strong. The precise relation between the different measures remains to be determined.

**Table 6:** **Spearman correlation coefficients *r* for the measures studied (*p* < 0.001 for all comparisons)**.

	**Self-similarity**	**Complexity**	**Anisotropy**	**Fourier slope**	**Fractal dimension**
Self-similarity	–	0.73	−0.53	0.52	0.60
Complexity	0.73	–	−0.45	0.66	0.82
Anisotropy	−0.53	−0.45	–	−0.59	−0.45
Fourier slope	0.52	0.66	−0.59	–	0.63
Fractal dimension	0.60	0.82	−0.45	0.63	–

An intermediate level of complexity has been previously linked to aesthetic perception. Berlyne (1974) described a u-shaped dependence of the hedonic value of aesthetic stimuli on complexity (or information content). He postulated that this curve could be explained by the different degrees of activation of two antagonistic systems in the human brain, a reward system and an aversion system. For visually pleasing stimuli, the dependence of beauty on complexity is not straightforward, partially because physical measures of complexity differ between studies (Forsythe et al., 2011) and there is a general lack of well-controlled studies that manipulate complexity in such images, with few exceptions (Jacobsen and Hofel, 2002; Taylor et al., 2005; Forsythe et al., 2011). Nevertheless, the present results support the notion that the different categories of visually pleasing images have an intermediate degree of complexity in general.

Furthermore, the present results indicate that the PHOG-derived self-similarity measure used by us attains a similar degree in advertisements, artworks and natural scenes. The Fourier slope is not highly correlated with the self-similarity measure (Table 6). The Fourier slope of subsets of monochrome artworks and monochrome images of natural scenes share a slope value of around −2 (Graham and Field, 2007; Redies et al., 2007a,b; Alvarez-Ramirez et al., 2008), but the slope value of colored artworks converted to grayscale images is much lower (−2.8 in the present study; -2.9 for colored art portraits in the study by Redies et al., 2007b). However, monochrome artworks are not equivalent to grayscale versions of colored paintings because color plays a pivotal role in aesthetic appreciation (Palmer et al., 2013). Consequently, conversion of colored artworks to grayscale version may destroy their aesthetic appeal.

Median anisotropy is higher for advertisements than for aesthetic artworks and natural scenes. This finding may relate to the presence of vertical text and image divisions along cardinal (horizontal and vertical) orientations in advertisements. Strikingly, all image categories studied are significantly more anisotropic on average than artworks, except for the natural categories (natural scenes and branches), although there is some degree of overlap (Figures 5C,D). A relatively low degree of anisotropy of artworks has been observed before in other studies (Koch et al., 2010; Redies et al., 2012; Melmer et al., 2013). This result is remarkable because, conceivably, artists can also produce highly anisotropic images. Note that specific natural patterns, such as lichen growth patterns and branches, can have even lower anisotropy values (Redies et al., 2012). In how far the responses of the visual system to isotropic and anisotropic visual stimuli relate to aesthetic perception is unclear at present.

The separation of the different image categories is especially clear in the scatter plots of anisotropy vs. the Birkhoff-like measure, which we defined as self-similarity divided by complexity (Figure 5D). Each image category, including advertisements and artworks, is characterized by a specific pattern of values defined by the three measures. Whether this pattern is required or sufficient for aesthetic perception remains to be studied.

### Dependence of aesthetic measures on advertisement content

Within the seven subcategories of advertisements, differences were observed, some of which were anticipated. For example, single-product advertisements are generally less complex than leaflets that promote multiple products on each page. Magazine covers, which contain a relatively large amount of printed text, are also more complex. It is likely that the high complexity of leaflets and magazine covers relates to the fact that they display multiple visual elements that can each attract attention separately (e.g., headings, text banners and price information) whereas single-product advertisements are the result of a more integrated visual composition that encompasses the entire image. As a consequence, in single-product advertisements, the appeal of the product may be carried by the global appearance of the entire image and not by its parts, such as in leaflets. In this respect, single-product advertisements may resemble artworks. For this reason, it is perhaps not surprising that the two types of images have similar statistical image properties.

Like complexity, self-similarity differs significantly between the various types of advertisements. Nevertheless, single-product advertisements and show windows exhibit a similar degree of self-similarity in general, when compared to the other image categories. Also, differences in anisotropy between single-product advertisements are relatively small (Figure 4, Table 4).

In conclusion, for the single-product advertisements studied, image properties are similar irrespective of the type of product shown. Whether and by what perceptual mechanisms these properties lead to a higher efficiency in promoting products remains to be studied.

### Images of architecture and their “biophilic” structure

As expected, self-similarity of buildings and facades is relatively high (Figure 4), possibly because they are composed of repetitive visual elements, such as windows and architectural ornaments. In contrast, urban scenes contain elements of more diverse forms (e.g., cars, trees, street surfaces and buildings) and are less self-similar. The complexity of architectural images is higher than in artworks in general. Compared to natural scenes, images of buildings and facades tend to be more complex while urban scenes share a similar degree of complexity. The relatively high degree of anisotropy in architectural images was anticipated because cardinal orientations are prominent in most types of architecture and urban scenes, as demonstrated before (Oliva and Torralba, 2006).

Natural environments are thought to be particularly rich in stress-reducing, restorative elements (Kaplan, 1995). It has been proposed that humans possess a visual preference for natural, fractal-like patterns in architecture and urban scenes (“biophilia” hypothesis; see Introduction). The overall similarities between architectural images and natural scenes in self-similarity and complexity support this idea. However, anisotropy in our set of architectural images is much higher than in natural scenes and natural patterns, such as branches. Most likely, anisotropy is lower in specific types of architecture that prominently feature oblique orientations, for example the buildings by Antoni Gaudí or Friedensreich Hundertwasser. Other styles of architecture, for example the Bauhaus style, are characterized by a conspicuous lack of oblique orientations (Salingaros, 1999), leading to high anisotropy. The role of anisotropy in architectural aesthetics therefore remains unclear.

### Conclusion and hypothesis

The present study demonstrates that images of advertisements are characterized by a specific combination of higher-order image properties (high self-similarity, intermediate complexity and intermediate anisotropy) on average. For single-product advertisements, these properties do not depend on the type of product promoted. We hypothesize that the processing of such higher-order image features can be fast and may be mediated at a lower level of the visual system, similar to gist perception of scenes (Torralba and Oliva, 2003; Oliva and Torralba, 2006), possibly occurring even before the content of the advertisements (e.g., the depicted object, brand name etc.) is recognized. The degree of self-similarity and complexity in advertisements is close (but is not identical) to that of artworks and natural scenes. It has been shown that higher-order image properties similar to natural scenes allow an efficient processing in the visual system (Parraga et al., 2000; Simoncelli and Olshausen, 2001). This idea has been extended to visual artworks (Redies, 2007; Graham and Redies, 2010). Here, we speculate that the idea may also apply to print advertising, at least to some degree. Possibly, specific higher-order image properties enhance the visual effectiveness of advertisements. At higher levels of (cognitive) processing, the effectiveness of advertisements also depends on other factors, such as the psychological condition of the observer and the real or stimulated demand for the product. Interestingly, images of architecture share statistical properties with advertisements to a large extent. It remains to be investigated whether any of these image properties (or a combination thereof) plays a causative role in judging visual stimuli as perceptually pleasing.

### Conflict of interest statement

The authors declare that the research was conducted in the absence of any commercial or financial relationships that could be construed as a potential conflict of interest.

## Figures and Tables

**Figure A1:**
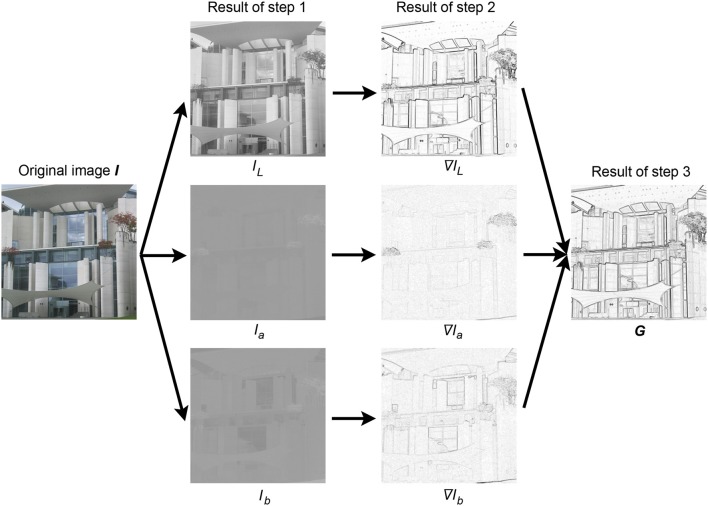
**Method to obtain the gradient image ***G*****. From left to right, the original image ***I***, the image divided into the three channels of the Lab color space (*I_*L*_*, *I*_*a*_ and *I*_*b*_), their respective gradient images (∇ ***I***_***L***_, ∇ ***I***_***a***_, and ∇ ***I***_***b***_), and the resulting gradient image ***G*** are displayed. The brightness scale for the images in the two columns on the right hand side was expanded so the brightness changes can be seen more readily.

**Figure A2:**
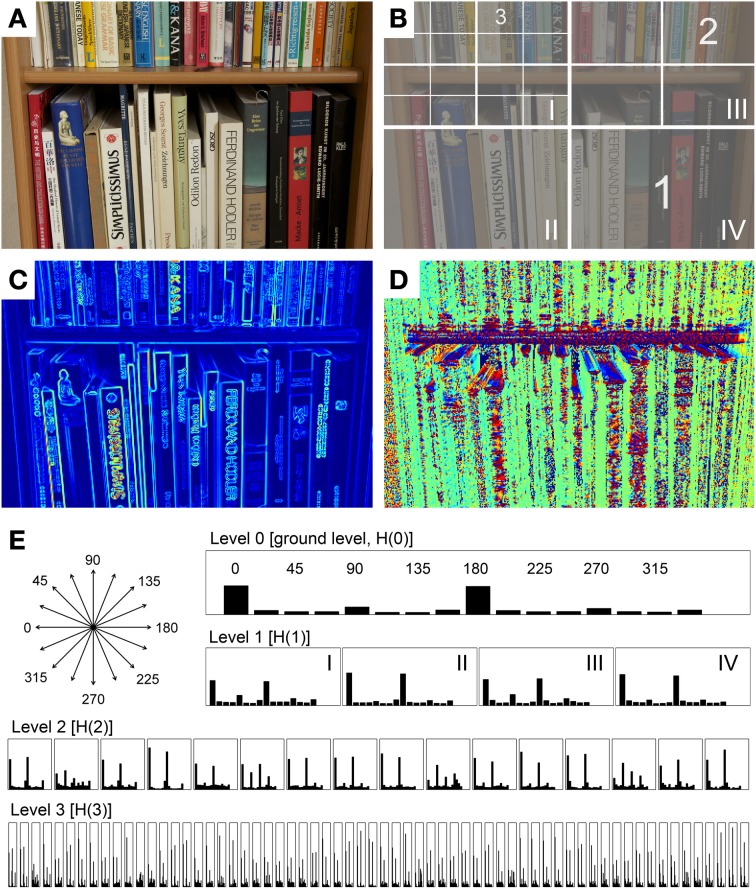
**Calculation of self-similarity by the PHOG method**. **(A)** Original photograph. Note the prominence of vertical orientations in the image. **(B)** Schematic diagram of the different levels of spatial resolution (1–3), superimposed onto the photograph shown in **(A)**. Level 1 contains four sections, level 2 sixteen sections, and level 3 64 sections. **(C)** Image of gradient strength, indicated by rainbow pseudocolor coding. **(D)** Gradient orientation image. Colors indicate different orientations (e.g., *red* for horizontal orientations, and *green* for vertical orientations). **(E)** HOG features at the different levels of spatial resolution (levels 0–3). The orientations of the 16 bins used for calculating the HOG features are displayed at the upper left side of the panel. The Arabic numerals indicate the binned orientations [see panel **(B)**]. The Roman numerals in the level 1 histogram indicate the subsections [compare to level 1 in **(B)**].

## Appendix A

This appendix gives an overview of the method that was used to calculate values for self-similarity, complexity and anisotropy (Amirshahi et al., 2012; Redies et al., 2012). The method is based on the Pyramid Histogram of Oriented Gradients (PHOG) approach. PHOG descriptors are spatial shape descriptors that were originally introduced by Bosch et al. (2007) for image classification. They are global feature vectors based on a pyramidal subdivision of an image into sub-images, for which Histograms of Oriented Gradient (HOG; Dalal and Triggs, 2005) are computed. In computer vision, such a data structure is called a *quadtree*.

In this approach, the following steps are taken to calculate a gradient image ***G*** (shown in Figure A1) for a given color image ***I***.

1. In the Lab color space, the image ***I*** is separated into its three channels (*I*_*L*_, *I*_*a*_, and *I*_*b*_). The L channel represents the luminance. The a and b channels are the red-green and blue-yellow opponent channels, respectively.
2. The gradient image is then calculated for each of the three images, which we have introduced in step 1 and are represented as ∇ *I*_*L*_, ∇ *I*_*a*_, and ∇ *I*_*b*_ in Figure A1. In this work, the gradient is calculated using the Matlab function *gradient*.
3. For each pixel in the three gradient images calculated in step 2, the maximum value among the three available values is selected and placed in a new image, ***G***. From here on, we will refer to ***G*** as the gradient image. The following equation represents this approach in another way:
  $$G(x,y)=\max\left(\left\|I_{L}(x,y)\right\|,\left\|I_{a}(x,y)\right\|,\left\|I_{b}(x,y)\right\|\right)$$

As an example, Figure A2C shows the gradient image ***G*** of the photograph displayed in Figure A2A.

Next, the HOG features are calculated (Dalal and Triggs, 2005). HOG is based on the orientation of gradients in an image. Using image ***G***, we separate the orientations of the gradients (Figure A2D) into *n* bins resulting in a HOG feature

$$h=\left(h_{1},h_{2},\cdots ,h_{\text{n}}\right)$$

of size *n*. Although the value for *n* and the range of orientations could be any arbitrary number, using 8 or 16 bins with 180 or 360 degrees is common. In the present study, we used 16 bins covering 360 degrees (Figure A2E). To obtain the HOGs, the strength of all gradients is calculated for each bin. As the last step in the HOG calculation, the histogram values are normalized so that the sum of the values for all 16 bins is one.

The self-similarity measure is obtained by calculating the HOG features for each sub-image of the quadtree (Figure A2B) as follows:

1. First, we calculate the HOG feature for the entire gradient image at level 0 (ground level).
2. Next, we divide the image into four equal sub-sections and calculate HOG features for each sub-section (level 1).
3. Each sub-section introduced at level 1 is divided again into four equal sub-sections (level 2) and the HOG features are calculated for the resulting 16 sub-sections.
4. The division and calculation can be continued as long as desired. In the present study, the highest level was level 3 (64 sub-sections). Higher levels result in even smaller sections with increasingly uniform distributions of luminance and fewer gradients, producing unstable results (Amirshahi et al., 2012).

The results of all HOG features at the individual levels of the quadtree (step 1–4) are concatenated to a features vector being the PHOG representation of the image. An example of the underlying histograms is depicted in Figure A2E.

To calculate the degree of self-similarity of an image, the Histogram Intersection Kernel (HIK; Barla et al., 2002) is used

$$\text{HIK}\left(h,h^{\prime }\right)=\sum_{i=1}^{n}\min\left(h_{i},h\prime_{i}\right)$$

to determine how similar two HOG features are. In the above equation, ***h*** and ***h***' represents two sets of HOG vectors for two sub-sections in the image and h_i_ represents the value in the *i*th bin in ***h***. The range of HIK is between 0 and 1. As it can be seen from the equation, the HIK function will result in the value of one in the case of two matching HOG features. The value of zero is reached if the entries for h_i_ or h'_i_ is zero for all bins. The self-similarity for image ***I*** at any level *L* is calculated by

$$m_{ss}(I,L)=\text{median}\left(HIK\left(h(S),h(N(S))\right)\right).$$

In this equation, **h**(*N*(*S*)) corresponds to the nod (section) in the quadtree of the section, to which sub-section *S* is compared (parent section, ground section or neighboring sections; see Figure 3), and ***h*** represents the HOG vectors. Sample values for the self-similarity measure are given in the main text. By selecting the median value among the different values, we avoid taking the overshoots into account.

The mean of all gradient strengths in the gradient image ***G*** serves us as a measure of the complexity of the image in the present study. A uniform original image with small changes in pixel values would result in a gradient image of low mean values (low complexity) while an image with large changes would result in a gradient image of high mean values (high complexity). To calculate complexity for image *I*,

$$m_{Co}(I)=\frac{1}{N\cdot M}\sum_{\left(x,y\right)}G(x,y)$$

the mean value over the gradient image, ***G*** is calculated. In this equation, *N* and *M* are the height and width of image ***I***, respectively.

The HOG approach also allows deriving a measure for how different the strengths of the gradients are across orientations in an image (anisotropy). Low anisotropy means that the strengths of the orientations are uniform across orientations and high anisotropy means that orientations differ in their overall prominence. For example, in the photograph shown in Figure A2A, anisotropy is high because vertical orientations (0 and 180°) are more prominent than the other orientations. As a measure of anisotropy, we calculate

$$m_{AnI}(I,L)=\sigma (H(L)),$$

over the HOG feature entries for image ***I*** at the last level (*L* = 3 in our calculation). In this equation, *H*(*L*) corresponds to the HOG feature at level *L* and σ is the standard deviation,

$$\sigma (H(L))=\sqrt{\frac{1}{m}\sum_{i=1}^{m}\left(h_{\text{i}}-\mu_{H(L)}\right)}.$$

In this equation, μ_*H*(*L*)_ corresponds to the mean value of all the bins at level *L*, while *m* represents the number of bins at level *L*. If each section of the images is divided to *sec* new subsections,

$$m={\left(\text{sec}\right)}^{l}$$

at level *l*. An image with a high degree of anisotropy will result in values that change a lot over the feature entries, while an image with a low degree of anisotropy will result in feature values that are approximately equal.

According to Birkhoff (1933), the aesthetic value depends on the ratio of order and complexity. Following this idea, we substituted order by self-similarity to obtain a Birkhoff-like measure, as described in Redies et al. (2012). This measure was calculated for level 3 based on the ground approach.
